# Extracellular vesicular microRNAs and cardiac hypertrophy

**DOI:** 10.3389/fendo.2024.1444940

**Published:** 2025-01-09

**Authors:** Hai Hu, Xiulian Wang, Hui Yu, Zhanli Wang

**Affiliations:** ^1^ Inner Mongolia Key Laboratory of Disease-Related Biomarkers, The Second Affiliated Hospital, Baotou Medical College, Baotou, China; ^2^ School of Basic Medicine, Baotou Medical College, Baotou, China

**Keywords:** cardiac hypertrophy, extracellular vesicle, microRNA, inflammation, regulatory mechanism

## Abstract

Cardiac hypertrophy is an adaptive response to pressure or volume overload such as hypertension and ischemic heart diseases. Sustained cardiac hypertrophy eventually leads to heart failure. The pathophysiological alterations of hypertrophy are complex, involving both cellular and molecular systems. Understanding the molecular events that inhibit or repress cardiac hypertrophy may help identify novel therapeutic strategies. Increasing evidence has indicated that extracellular vesicle (EV)-derived microRNAs (miRNAs) play a significant role in the development and progression of cardiac hypertrophy. In this review, we briefly review recent advancements in EV research, especially on biogenesis, cargoes and its role in cardiac hypertrophy. We then describe the latest findings regarding EV-derived miRNAs, highlighting their functions and regulatory mechanisms in cardiac hypertrophy. Finally, the potential role of EV-derived miRNAs as targets in the diagnosis and treatment of cardiac hypertrophy will be discussed.

## 1 Introduction

Cardiac hypertrophy serves as a compensatory mechanism to address the reduced cardiac output and represents a restricted form of adjustment in response to increased cardiac stress. Pathological cardiac hypertrophy as a well-recognized risk factor for cardiovascular diseases (CVDs) (1, 2), generally accompanies cardiomyocyte enlargement and ventricular remodeling (3, 4). Subsequently, long-term pathological factors contributed to extensive cardiac remodeling, including the development of cardiac fibrosis, hypertrophy, and ultimately heart failure (5). The heart is composed of a diverse combination of cell types, such as cardiomyocytes, fibroblasts, endocardial and epicardial cells, inflammatory cells, and immune cells. These cellular components within the cardiac system communicate extensively to ensure proper cardiac function through direct cellular interaction or paracrine signaling (6). Previous studies have documented the existence of intercellular communication between cardiomyocytes and noncardiomyocytes during the development of cardiac hypertrophy (7–10). This intercellular communication mainly occurs through direct cell-to-cell interaction, specific molecules, or extracellular vesicles (EVs) (11–13).

EVs, which are membranous particles composed of lipid bilayers, are released by various cell types in organisms ranging from bacteria to humans and plants (14–16). Based on their dimensions, EVs have been categorized into three groups comprising exosomes (sizes of 30–150 nm), microvesicles (sizes of 100–1000 nm), and apoptotic bodies (sizes of 50 nm–10 μm) (17, 18). Being the primary classifications, exosomes and microvesicles share the same membrane orientation that is identical to the cell surface, but they vary in terms of size and cargo compositions (RNAs, proteins, lipids, or metabolites) (19). The composition of these functional components may vary based on the cellular source and specific pathophysiological conditions during EV packaging and release (20). EVs have been employed as potential signaling mediators, biomarkers, and potential therapeutic agents for cardiovascular physiology and pathology (21). The abundance of evidence suggests that EVs play a pivotal role in various cardiovascular processes, encompassing both physiological and pathological aspects. These include the regulation of angiogenesis and blood pressure, cardiomyocyte hypertrophy, apoptosis/survival, and cardiac fibrosis (22–26). In the past ten years, EVs have gained increasing recognition for their potential roles as important autocrine and paracrine intercellular communicators. They deliver molecular cargoes from source cells to neighboring cells or even to distant organs and influence the programming of the cardiac microenvironment (27, 28). MicroRNAs (miRNAs) are small noncoding RNAs, which are 22 to 25 nucleotides in length. They can bind to the 3′ untranslated regions of their target messenger RNAs (mRNAs), leading to mRNA degradation or translational repression (29). Direct regulation by miRNAs occurs in over 60% of the protein-coding genes (30). Furthermore, the miRNAs present in cardiovascular exosomes have been shown to regulate target genes within recipient cardiac cells, thereby influencing both physiological and pathophysiological processes (31). In this review, we summarize the current understanding of the roles of EVs in cardiac diseases, with a particular emphasis on pathological cardiac hypertrophy. Specifically, we will attempt to provide an overview of the potential values of EVs-derived miRNAs in the field of pathological cardiac hypertrophy as therapeutic agents and biomarkers.

## 2 Biogenesis of EVs

The generation of exosomes is linked to the endosomal network. Exosomes are generated through the inward budding of the endosome’s limiting membrane into its lumen, as intraluminal vesicles (ILV) (32, 33). Further continued inward budding of the endosomal membrane leads to the formation of the multivesicular body (MVB). Finally, the MVBs merge with the cellular membrane and release the ILVs as exosomes into the extracellular environment. Alternatively, the MVBs can combine with lysosomes inside the cell and release the ILVs for either degradation or recycling within the cellular context. Both ESCRT-dependent and ESCRT-independent mechanisms contribute to the packaging of cargoes into endosomes and the formation of exosomes in multivesicular bodies (MVBs) (34–36). The ESCRT-dependent pathway, consisting of subcomplexes ESCRT-0, ESCRT-I, ESCRT-II, and ESCRT-III, is responsible for the sorting of various nucleic acids and proteins. ESCRT-dependent pathway also has been shown to regulate the inward budding of intraluminal vesicles (ILVs) into the lumen of endosomes and the final forming of MVBs (37, 38). An alternative ESCRT-independent pathway requires the involvement of the cellular Golgi apparatus and the tetraspanin family proteins (CD63, CD81, CD82 and CD9), facilitating the formation of membrane microdomains and sorting cargo to MVBs (39). Although exosome biogenesis is commonly classified as ESCRT-dependent or ESCRT-independent, these pathways may not be entirely distinct. Whereas, the generation of microvesicles occurs within the extracellular space through direct outward budding of the cell’s plasma membrane, which exhibits key characteristics of stepwise vesiculation observed in exosome formation and employs the same machinery (40). Exosomes and microvesicles are considered dynamic carriers of cellular communication owing to their release and uptake by living cells. The formation of apoptotic bodies, on the contrary, is generated from the cell membrane, as a result of cellular disassembly during the apoptosis (41).

## 3 EV cargoes and intercellular communication

EVs transport both membrane-bound and soluble cargoes, which are characterized by distinct compositions of lipids, proteins, and nucleic acids. The lipid composition of their membranes exhibits similarities to raft microdomains, displaying higher levels of cholesterol, sphingomyelin, phosphatidylserine, and ceramide compared to the plasma membrane (42). It is widely recognized that EVs possess a rich assortment of proteins, encompassing those derived from the plasma membrane, cytosol, cytoskeleton, and proteins engaged in vesicle trafficking (43). Exosomal protein markers encompass the ESCRT machinery proteins (Alix, TSG101, and VSP40), Syntenin-1 (44), heat-shock proteins (Hsp20, Hsp60, Hsp70, and Hsp90), transmembrane tetraspanins (CD62, CD63, CD9, and CD81) (45), and blood circulating EVs-derived cytokines (IL-1b, IL-6, TGF-β, and TNF) (46, 47). EV-carried cell-type specific proteins, especially membrane surface proteins, have been very useful for identifying and isolating cell-type specific EVs (48). The cargo nucleic acids possess significant potential as efficient facilitators of inter-tissue communication, despite indications that the levels of RNAs in EVs are generally modest (49). The transfer of miRNAs and mRNAs to target cells through EV-mediated mechanisms has been extensively acknowledged for its functional importance (50, 51). Other types of RNA in EVs are tRNAs, tRNA fragments, fragmented mRNAs, long non-coding RNAs (lncRNAs), and ribosomal RNAs, mitochondrial RNAs. EVs traverse the extracellular fluid, attach to the matrix surrounding cells and intercellular junctions, migrate towards adjacent tissue regions, or gain access to major body fluids to reach distant organs (52, 53). In recent years, EVs have been widely acknowledged for their crucial involvement in intercellular signaling, facilitating the transfer of various biomolecules such as lipids, DNA, mRNA, miRNA, siRNA, and lncRNA to recipient cells. Generally, the transfer of biological signals from EVs to target cells occurs through various mechanisms that rely on the composition of proteins and lipids present on both the surfaces of EVs and target cells. The interaction between EVs and target cells occurs via membrane-bound ligand-receptor pairs, which subsequently initiate intracellular signaling pathways. Target cells can internalize EVs via diverse endocytic pathways, including macropinocytosis, phagocytosis, clathrin-mediated endocytosis, caveolin-dependent endocytosis, and caveolin-independent endocytosis (54).

## 4 EVs in cardiac hypertrophy

Pathological cardiac hypertrophy is distinguished by the cardiomyocytes’ enlargement, the existence of interstitial fibrosis, and insufficient blood supply to the heart. These factors collectively contribute to the development of cardiac dilation, impaired systolic and diastolic function, and ultimately heart failure (3, 4). Cardiac hypertrophy is accompanied by disrupted electrophysiology and metabolism during adverse remodeling. While cardiac fibroblasts transform into activated myofibroblasts, exhibiting proliferation and excessive production of extracellular matrix (3, 55).

EVs play important roles in intercellular communication among nearby and distant cells, including cardiac cells. They have been implicated in the regulation of various processes such as cardiomyocyte hypertrophy, apoptosis, cardiac fibrosis, and angiogenesis (56–58). EVs can be separated from various cell types present in the heart, including cardiac fibroblasts, cardiomyocytes, endothelial cells, vascular smooth muscle cells, and Mesenchymal stem cells (MSCs) (59–71).

As the predominant cell type in the heart, Cardiomyocytes play crucial roles in various pathological processes, including cardiac hypertrophy, angiogenesis, cardiac fibrosis, autophagy, and apoptosis (72). The release of specific exosomes by cardiomyocytes has been investigated in the context of pathological cardiac hypertrophy (73, 74). Furthermore, the production and release of EVs originating from cardiomyocytes are impacted by various factors. For example, Hsp20 facilitated the initiation of exosome formation and their release from cardiomyocytes through its interaction with Tsg101, a key regulator of exosome biogenesis (75). Besides, cardiomyocytes can selectively modify the contents of their exosomes and enhance the exosomes’ release in response to various stressors such as glucose deficiency, oxygen deprivation, inflammatory conditions, physical trauma, or elevated angiotensin II levels (76–78). Specific proteins, including two members of the heat shock protein family (Hsp20 and Hsp90), can be secreted by cardiomyocytes to facilitate the restoration of cardiac function in the context of pathological cardiac hypertrophy (79–82). One research study demonstrated that diabetic cardiomyocytes can release exosomes with lower levels of Hsp20. Conversely, the upregulation of Hsp20 has been found to mitigate cardiac dysfunction, hypertrophy, apoptosis in cardiomyocytes, fibrosis in the heart tissue, and decreased density of microvessels induced by STZ (75). Additionally, previous research has indicated that Hsp90 plays a pivotal role in modulating ventricular hypertrophy by activating the MAPK pathway, NF-κB pathway, STAT-3 pathway, and stabilizing HIF-1 alpha. Exosomal Hsp90 derived from cardiomyocytes, along with secreted IL-6, participates in the activation of STAT-3 signaling within cardiac fibroblasts, leading to enhanced synthesis and accumulation of collagen during cardiac hypertrophy (81).

On the other hand, exosomes derived from fibroblasts have a crucial function in facilitating communication between cardiomyocytes and fibroblasts throughout the hypertrophic procedure (83–87). The administration of angiotensin II to fibroblasts was found to enhance the release of exosomes, thereby inducing cardiomyocyte hypertrophy *in vitro* through the activation of Akt and mitogen-activated protein kinases (MAPKs), as well as increased expression of the renin-angiotensin system (RAS) in cardiomyocytes (63). The study mentioned above shows that the fibroblast-derived exosome proteins osteopontin (Spp1) and epidermal growth factor receptor (EGFR) can trigger the PI3K/Akt and MAPK pathways, resulting in the upregulation of RAS in cardiomyocytes.

## 5 Biogenesis of miRNAs

MiRNAs, as small endogenous oligonucleotides ranging from 21 to 25 nucleotides in length, play a crucial role in the post-transcriptional regulation of genes by binding to or inhibiting target mRNAs (88, 89). MiRNAs genes are initially transcribed by RNA polymerase II as primary miRNAs (pri-miRNA), the long transcripts containing either non-coding or coding hairpins (90). Then, pri-miRNAs are enzymatically processed to form precursor miRNA (pre-miRNA) by the functional Microprocessor complex, comprising RNase III Drosha and DiGeorge syndrome critical region gene 8 (DGCR8) (91). The pre-miRNA is subsequently exported to the cytoplasm by exportin 5 (Exp-5) and is finally processed into a mature miRNA by the RNase III Dicer (91). Non-canonical miRNA biogenesis, including the Drosha-independent and the Dicer-independent pathways, are also observed (92).

It is well known that miRNA-mediated transcriptional and post-transcriptional gene regulation within the cells. Additionally, miRNAs can be released from cells into the circulation in EVs. Recently, Garcia-Martin et al. reported that that miRNAs possess sorting sequences determined their EV secretion or cellular retention (93). However, the mechanisms by which miRNAs are secreted into EVs or retained in cells not well understood and require further investigation.

## 6 EV-derived miRNAs in cardiac hypertrophy

**Figure 1:**
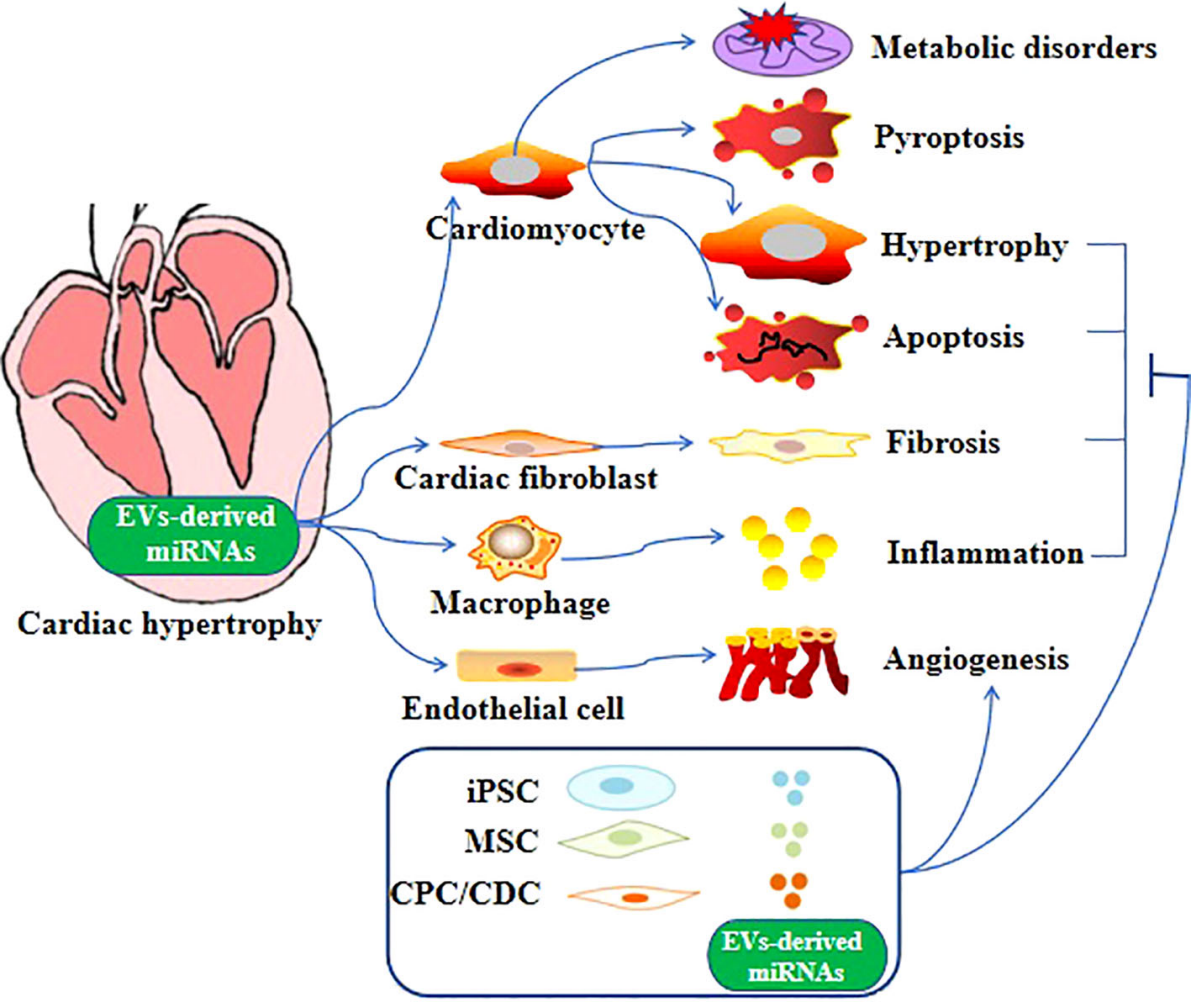
Role of EV-derived miRNAs in cardiac hypertrophy. Different EV-derived miRNAs cause cardiac hypertrophy through different mechanisms and even multiple mechanisms.

Altered expression of miRNAs has been linked to cardiovascular disease conditions, such as heart failure and cardiac hypertrophy (94–98). For example, Bernardo et al. reported that inhibition of miR-34a could attenuate cardiac dysfunction in a mouse model with pre-existing pathological hypertrophy (99). In addition, miRNA expression analysis in the human heart found a correlation between downregulated miRNAs in cardiospheres/cardiosphere-derived cells and patient age (100). Apart from these proteins mentioned above aforementioned proteins, specialized miRNAs-containing EVs can be released by cardiomyocytes to facilitate the restoration of cardiac function in cases of pathological hypertrophy (101–103). Furthermore, compelling evidence suggests that in pathological conditions, miRNAs are secreted from various cell types, including fibroblasts, endothelial cells, stem cells, and immune cells (104). These miRNAs are then transferred to cardiomyocytes through EV-mediated trafficking (**Figure 1**), directly promoting the hypertrophic growth of cardiomyocytes (86, 105). EV-derived miRNAs that have been found to regulate cardiac hypertrophy are shown in **Table 1**.

**Table 1:** EV-derived miRNAs that have been found to regulate cardiac hypertrophy.

EV-derived miRNA	Cells responsible for secretion	Recipient cells	Targets	Functions	References
miR-21	Tubular epithelial cells	Cardiomyocytes	SORBS2, ENH	Promotes cardiomyocytes hypertrophy	(106)
miR-21-3p	Cardiac fibroblasts	Cardiomyocytes	SORBS2,PDLIM5	Promotes cardiomyocytes hypertrophy	(59)
miR-27a	Cardiac fibroblasts	Cardiomyocytes	Nrf2	Increases oxidative stress and expression of genes associated with hypertrophy	(112)
miR-27a*	Cardiac fibroblasts	Cardiomyocytes	Nrf2/ARE,PDLIM5	Promotes cardiomyocytes hypertrophy	(113)
	Cells from pericardial adipose tissue	Cardiomyocytes	AMPK/PINK1/Parkin signaling	Promotes cardiomyocytes hypertrophy	(115)
miR-29a	MSCs	Cardiomyocytes, Cardiac fibroblasts	Unknown	Protects cardiomyocytes against pathological hypertrophy	(116)
	Cardiomyocytes	CMEC	VEGFA	Inhibits the angiogenic ability of CMECs	(117)
miR-126	Endothelial cells	Cardiomyocytes	VCAM-1, MCP-1	Inhibits cardiomyocytes hypertrophy	(119)
	Diabetic cardiomyocytes	Endothelial cell	Unknown	Impaired angiogenesis and cardiac functions	(120)
miR-146	Cardiac fibroblasts	Cardiomyocytes	SUMO1	Induces cardiac dysfunction	(86)
	Endothelial cells, cardiac fibroblasts	Cardiomyocytes	Erbb4, Nras, Notch1, Irak1	Impaired metabolism and function of cardiomyocytes	(124)
	CDCs	Unknown	Unknown	Improves cardiac function and reduced myocardial fibrosis	(125)
miR-155	Macrophages	Cardiomyocytes	FoxO3a	Promotes cardiomyocytes pyroptosis	(127)
	Cardiomyocytes	Macrophages	MAPK	Promotes inflammation in macrophages	(128)
	Cardiomyocytes	Unknown	Jarid2	Promotes cardiomyocytes hypertrophy	(129)
miR-181a	Cardiomyocytes	Unknown	Unknown	Promotes cardiomyocytes hypertrophy, increases myocardial fibrosis	(103)
miR-200a	Adipocytes	Cardiomyocytes	TSC1, mTOR	Promotes cardiomyocytes hypertrophy	(133)
miR-133a	CPCs	NRCMs	Bim, Bmf	Reduces fibrosis and hypertrophy	(135)
miR-217	Cardiomyocytes	Cardiac fibroblasts	PTEN	Enhance fibroblast proliferation, increases myocardial fibrosis	(101)
miR-22	CF-iPSCs	Embryoid bodies	Unknown	Regulates cardiac hypertrophy and remodeling	(134)
miR-27b	Cardiomyocytes	Unknown	TGF-β1-PPARγ pathway	Promotes cardiomyocytes hypertrophy	(137)
miR-212/-132	Cardiac fibroblasts	Cardiomyocytes	ANG II-AT1R axis	Promotes cardiomyocytes hypertrophy	(138)
miR-1, miR-222, and miR-208a	Skeletal muscle cells	Cardiomyocytes	Unknown	Promotes cardiomyocytes hypertrophy	(138)
miR-199a	Unknown	Unknown	mTOR	Promotes cardiomyocytes hypertrophy	(132)
miRNA-148a	CDCs	Cardiomyocytes	STAT3/ERK1/2/AKT signaling pathway	Improves cardiac function and remodeling	(141)

CDCs, cardiosphere-derived cells; CF-iPSCs, induced pluripotent stem cells derived from cardiac fibroblasts; CMEC, cardiac microvascular endothelial cell; CPCs, cardiac progenitor cells; MSC, mesenchymal stem cell; NRCMs, neonatal rat cardiomyocytes; PBMCs, peripheral blood mono-nuclear cells.

### 6.1 miR-21

It has been found that EVs can be transferred from tubular epithelial cells to cardiomyocytes and facilitate the development of cardiomyocyte hypertrophy when renal tubular cells are treated with TGF-b1 (106). However, this effect could be eliminated by a miR-21 inhibitor, suggesting that miR-21 derived from EVs can be transmitted to recipient cardiomyocytes and contribute to cardiomyocyte hypertrophy. Through further investigation, Jia and colleagues have substantiated that the presence of miR-21 in EVs can induce cardiomyocyte hypertrophy by targeting SORBS2 and ENH. Moreover, the investigation conducted by Chuppa et al. revealed that inhibition of miR-21 safeguards rats with 5/6 nephrectomy against the progression of left ventricular hypertrophy and deterioration in left ventricular function (107). It has also been found that the pericardial fluid of mice with induced hypertrophy through transverse aortic constriction exhibited an elevated level of subtypes of miR-21 (miR-21-3p, miR-21^*^) compared to sham-operated mice. Additionally, the inhibition of using miR-21-3p antagomir effectively prevented Ang II-induced cardiac hypertrophy (59). In their study, Bang et al. revealed the selective enrichment of miR-21-3p in fibroblast-derived exosomes and emphasized that factors such as temperature and actin dynamics are crucial for cardiomyocyte uptake of exosomes. Upon internalization into cardiomyocytes, the presence of miR-21-3p derived from exosomes resulted in a significant augmentation of cardiomyocyte cell size (108). Specifically, previous studies identified that exosomal miR-21-3p is transported into cardiomyocytes, where it targets SORBS2 and PDLIM5 (PDZ and LIM domain 5), contributing to cardiac hypertrophy (59, 109). However, many important questions remain elusive. For example, multiple types of pro-fibrotic cells are involved in the development of fibroblast during cardiomyopathy, it is therefore essential to explore the effects of exosomes on such a process of cardiac fibrosis (110). In addition, Yan et al. investigated the functional role of miR-21-3p in cardiac hypertrophy and demonstrated that its overexpression significantly mitigates TAC (transverse aortic constriction)-induced cardiac hypertrophy and Ang II-induced cardiac hypertrophy by suppressing HDAC8 expression to activate the Akt/Gsk3b pathway (111).

### 6.2 miR-27a

Previous studies by Tian et al. demonstrated that there was an increase in the expression levels of miRNA-27a in both infarcted heart tissue and circulating samples in rat models of chronic heart failure (112). They observed a significant upregulation of miR-27a in cultured cardiac fibroblasts following proinflammatory stimulation. miR-27a, as the predominant miRNA contained in cardiac fibroblast-derived EVs, was selectively incorporated into EVs and released into the surrounding environment. These EVs were then taken up by cardiomyocytes, leading to increased oxidative stress and expression of genes associated with hypertrophy in these cells by targeting Nrf2 signaling. In their subsequent investigations, Tian et al. made the discovery that miR-27a* (the passenger strand of miR-27a) functions as an additional star miRNA, which can exacerbate cardiac hypertrophy in a chronic heart failure model induced by myocardial infarction. In the presence of Ang II stimulation, cardiac fibroblasts exhibited an increase in miR-27a* expression (113). Subsequently, miR-27a* was selectively encapsulated within EVs and then released into the extracellular space. miR-27a*-enriched EVs were then internalized by cardiac myocytes, leading to the development of a hypertrophic phenotype through differentially targeting Nrf2/ARE signaling pathway and PDLIM5. It has also been discovered that the upregulation of miR-27a-3p can serve as a diagnostic indicator for cardiac hypertrophy in an *in vitro* model of cardiac hypertrophy by treating H9c2 cardiomyocytes with Ang II, as well as in an *in vivo* model achieved through chronic infusion of Ang II into mice. In addition, the upregulation of miR-27a-3p promotes cardiac hypertrophy by targeting NOVA1 (neurooncological ventral antigen 1) (114). Moreover, pericardial adipose tissue derived exosomal miR-27a-3p regulated myocardial hypertrophy and fibrosis via AMPK/PINK1/Parkin signaling (115).

### 6.3 miR-29

MiR-29a-containing exosomes derived from MSCs exhibited a significant cardioprotective effect by preventing pathological remodeling of the myocardium and maintaining heart function under conditions of pressure overload induced by TAC (116). In addition, cardiomyocyte-derived exosomal miR-29a inhibited the angiogenic ability of cardiac microvascular endothelial cell (CMEC) by targeting vascular endothelial growth factor (VEGFA) (117). Moreover, exosomal-miR-29 was identified as crucial targets from exercised hearts, which can facilitate cardiomyocyte growth and physiologic hypertrophy (118).

### 6.4 miR-126

MiR-126 plays a key role in modulating cardiac function. Chen et al. reported that low expression level of heart miR-126 was associated with cardiomyocyte hypertrophy, fibrosis, and inflammation. They further found that miR-126 was highly enriched in exosomes derived from endothelial cells. Meanwhile, exosome-enriched miR-126 inhibited cardiomyocyte hypertrophy by targeting vascular cell adhesion protein-1 (VCAM-1) and monocyte chemotactic protein-1 (MCP-1) (119). Moreover, previous study revealed that the expression of miR-126 was significantly decreased in exosomes derived from diabetic Goto-Kakizaki (GK) cardiomyocytes. Of interest, exosomal miR-126 derived from healthy cardiomyocytes can promoted normal angiogenesis (120). Previous study also observed that exosomal miR-126 derived from CD34+ peripheral blood mono-nuclear cells (PBMCs) exhibits pro-angiogenic effects. Moreover, pro-angiogenic effects of CD34+ PBMCs were blocked by anti-miRNA-126 treatment (120).

### 6.5 miR-146a

Previously recognized as a suppressor of innate immune responses, miR-146a exerts its regulatory effects through direct interactions with interleukin-1 receptor-associated kinase 1 (IRAK1) and TNF receptor-associated factor 6 (TRAF6) (121). Its roles in cardiac pathophysiology have been demonstrated to exhibit variability across different models of heart disease (122, 123). In a recent investigation, it was observed that miR-146a contributes to the development of cardiac dysfunction in maladaptive hypertrophy by reducing SUMO1 expression and regulating Ca^2+^ cycling. MiR-146a is released from transdifferentiated fibroblasts and subsequently transferred into cardiomyocytes through EV-mediated trafficking in the failing heart (86). In addition, miR-146a-enriched exosomes released from endothelial cells or fibroblasts contribute to improve contractile function in cardiomyocytes by targeting Erbb4, Notch1, and Irak1 (124). At the same time, cardiosphere-derived exosomal miR-146a-5p was associated with myocardial repair in pediatric dilated cardiomyopathy (125). Moreover, exosomal miR-146a could also serve as a potential biomarker for future heart failure diagnosis (126).

### 6.6 miR-155

In the case of the uremic heart, macrophage-derived miR-155–containing exosomes could enhance both pyroptosis and hypertrophy in the uremic cardiomyopathy model (105). Wang et al. found that miR-155 was synthesized and loaded into exosomes in increased infiltration of macrophages in a uremic heart. The fusion of exosomes with the plasma membrane results in the liberation of miR-155 into the cytosol, leading to the inhibition of translation for FoxO3a in cardiomyocytes. Ultimately, exosomes carrying miR-155 from macrophages were found to induce pyroptosis in cardiomyocytes and contribute to the development of uremic cardiomyopathy symptoms such as cardiac hypertrophy and fibrosis. This effect was achieved through direct inhibition of FoxO3a in uremic mice. Furthermore, inhibition of miR-155 using a specific inhibitor or the use of mice lacking miR-155 demonstrated that FoxO3a levels were partially recovered and reduced cardiomyocyte pyroptosis, hypertrophy, and fibrosis in uremic mice. Moreover, previous studies have suggested that exosomes derived from macrophages containing miR-155 act as a paracrine modulator of fibroblast proliferation and inflammation. This modulation results in the inhibition of fibroblast proliferation and an increase in fibroblast inflammation, ultimately leading to compromised cardiac repair following myocardial infarction (127). As it is widely acknowledged, the pathways leading to cardiac hypertrophy are closely linked to the pathogenesis of myocardial infarction. Intriguingly, the activation of the MAPK pathway may be triggered by miR-155-enriched exosomes derived from Ang II-induced hypertrophic cardiomyocytes, thereby inducing inflammation in macrophages (128). Taken together, microRNA-155, derived from EVs, plays a crucial role in facilitating communication between macrophages and fibroblasts or cardiomyocytes during the development of pathological cardiac hypertrophy. Relevant prior studies had shown that the activation of the Jaird2 signaling pathway in cardiomyocytes was facilitated by miR-155, leading to cardiac hypertrophy (129). Inhibition of endogenous Jarid2 partially ameliorated the impact of miR-155 deficiency in isolated cardiomyocytes.

### 6.7 miR-181a

A previous study conducted by Evgeniya et al. discovered that the administration of sacubitril/valsartan in human iCM (human-induced pluripotent stem cell-derived cardiomyocytes) led to a reduction in the expression of exosomal miR-181a. This decrease was found to contribute to the mitigation of myocardial fibrosis and hypertrophy (103). The downregulation of exosomal miR-181a was also observed by Evgeniya et al. in a rodent model of chronic myocardial infarction following sacubitril/valsartan treatment. Furthermore, the downregulation of exosomal miR-181a attenuates myocardial fibrosis and pathological hypertrophy, thereby restoring the chronic heart failure model in Sprague-Dawley rats. In Evgeniya et al. studies, the subsequent investigations utilized the chronic rodent myocardial injury model to explore the functionality of exosomal miR-181a. The validation was performed by employing miR-181a antagomir, which demonstrated favorable effects of exosomal miR-181a on cardiac function, volumes, and morphology. Other former relevant studies have demonstrated that miR-181a derived from cardiomyocytes possesses the potential to alleviate pathological hypertrophy in the heart by modulating multiple targets. For example, miR-181a as the newly discovered controller of cardiac remodeling, played a role in the regulation of autophagy, the p53-p21 pathway, PTEN/PI3K/AKT signaling, and the aldosterone-mineralocorticoid receptor (AldoMR) pathway in myocardial hypertrophy (130, 131).

### 6.8 miR-217

As a response to pathological hypertrophy, miR-217 was released from cardiomyocytes. The exosomes derived from cardiomyocytes, which contain miR-217, have been observed to enhance fibroblast proliferation and potentially contribute to cardiac fibrosis through the modulation of PTEN (132). Moreover, Nie et al. observed a significant upregulation of miR-217 in the cardiac tissues of patients with chronic heart failure. *In vivo*, overexpression of miR-217 exacerbated pressure overload-induced cardiac hypertrophy and dysfunction by repressing PTEN expression (101).

### 6.9 Other miRNA types

Fang et al. demonstrated that activation of PPARγ signaling in adipocytes enhanced the expression and secretion of miR-200a by employing cocultures comprising adipocytes and cardiomyocytes. Exosomal delivery of miR-200a from adipocytes to cardiomyocytes played a role in the development of cardiomyocyte hypertrophy by reducing TSC1 levels and subsequently activating mTOR (133). In addition, the expression of various miRNAs, particularly miR-22, a crucial controller of cardiac hypertrophy and remodeling, could be influenced by exosomes released from induced pluripotent stem cells derived from cardiac fibroblasts (CF-iPSCs) (134). Exosomal miR-133a released from ischemic cardiomyocytes or cardiac progenitor cells (CPCs) exerts inhibitory effects on hypertrophy (132, 135). Wu et al. showed that serum exosomal miR-92b-5p is a potential biomarker for the diagnosis of acute heart failure caused by dilated cardiomyopathy (136). In addition, another study has revealed that miR-27b participated in cardiac hypertrophy via the TGF-β1-PPARγ pathway (137). There is evidence to suggest that exosomal miR-212/-132 family may also induce cardiac hypertrophy (138, 139). Moreover, exosomal miR-1, miR-222, and miR-208a released from skeletal muscle were also involved in the regulation of cardiac hypertrophy (138). Exercise altered circulating exosomal miRNAs expression, including miR-486-5p, miR-215-5p, miR-941, and miR-151b. These exosomal miRNAs modulated cardiac hypertrophy through IGF-1 signaling (140). MiR-199a induces cardiac hypertrophy through mammalian target of rapamycin (mTOR) signaling pathway (132). Exosomal miRNA-148a from CDCs improves TAC-induced myocardial hypertrophy via down-regulation of GP130, leading to the inhibition of STAT3/ERK1/2/AKT signaling pathway (141). MiR-4731 overexpression promoted cardiac hypertrophy by targeting sirtuin 2 (SIRT2) (142). Exosomal miR 331-5p is a critical regulator of hypertrophy and fibrosis (143).

## 7 The summary and future outlook

Considerable advancements have been made in the past decade regarding our understanding of the biology associated with miRNAs derived from EVs and their pivotal role, particularly as proficient mediators of inter-tissue communication, in the physiology and pathological progression of cardiac hypertrophy (144–146). In this review, we focus on elucidating the biological functions and pivotal roles of extracellular EVs, encompassing their biogenesis, molecular cargoes, and facilitation of intercellular communication within the cardiovascular system. Furthermore, miRNAs derived from EVs exhibit promising potential in regulating pathological cardiac hypertrophy. These miRNAs-enriching EVs are involved in various functional behaviors of cardiac cells and intercellular communication, playing a critical role in the pathophysiological progression of cardiac hypertrophy.

The investigation into the biology of miRNAs derived from EVs holds significant potential for future therapeutic interventions aimed at cardioprotection, despite the limited research on EVs harboring miRNAs in cardiac hypertrophy. Further and in-depth research on the mechanism and role of miRNAs derived from EVs can contribute to the identification of novel interacting molecules and signal transduction pathways involved in cardiac hypertrophy, thereby offering innovative ideas and methodologies for its diagnosis and treatment. In the future, EV-derived miRNAs could potentially function as clinical indicators for cardiac hypertrophy owing to their distinctive inclusiveness. To establish EV-derived miRNAs as diagnostic or prognostic biomarkers in pathological cardiac hypertrophy, several technical challenges persist in this research field and more efforts are needed to conduct large randomized clinical trials.
